# Improving the Provision of Postoperative Driving Advice in Discharge Letters After Elective Inguinal Hernia Repair: A Quality Improvement Project

**DOI:** 10.7759/cureus.94630

**Published:** 2025-10-15

**Authors:** Anika Tahsin, Lilian Farren, Muhammad F Hayat, Saskia Chapman, Sylwia Osinska, James Phillips, Alireza Sherafat, Deepthi Haribaskaran, Wen Y Chung, Georgios Mistriotis

**Affiliations:** 1 General and Colorectal Surgery, Leicester Royal Infirmary, Leicester, GBR; 2 General Surgery, Leicester Royal Infirmary, Leicester, GBR

**Keywords:** clinician education, discharge letters, driving advice, inguinal hernia repair, patient safety, postoperative care, quality improvement

## Abstract

Background

Screening of discharge letters after inguinal hernia repair revealed that driving advice following inguinal hernia repair in the University Hospitals of Leicester (UHL) NHS Trust was often inconsistent or absent, which can pose a significant risk to patient safety. When advising patients on driving after groin hernia repair, there are a few factors to be considered, such as including research evidence and medicolegal literature, the development of a transient femoral nerve palsy and stiffness, individual variation postoperatively, and opioid prescription. The aim of this quality improvement project (QIP) was to ensure the provision of consistent, evidence-based postoperative driving advice aligning with the trust and the Royal College of Surgeons of England guidelines to patients following inguinal hernia repair in UHL.

Methodology

A retrospective audit of 42 patients’ discharge letters and the information provided to them by leaflets and verbal methods was conducted in September 2024, followed by a re-audit of 40 patients who had undergone inguinal hernia repair in November 2024. Alongside this, a survey of 50 clinicians was also conducted in September 2024, who work in general surgery, to assess their understanding and distribution of correct driving advice following inguinal hernia repair. This was followed by a survey of 30 clinicians in November 2024 to reassess their knowledge and distribution of driving advice after a teaching session, poster displays, and a reminder email to include driving advice in discharge letters following inguinal hernia repair surgery.

Results

This QIP demonstrated a significant improvement in patients receiving correct driving advice (from 14% to 43%) and in clinician understanding (from 33% to 90%). The first cycle of the QIP showed that 21 (50%) discharge letters did not contain driving advice, and 6 (14.3%) discharge letters contained correct advice. Overall, 10 (43%) of the contactable patients received information leaflets. Further, 11 (48%) of the contactable patients received verbal advice regarding safe driving. Overall, 17 (33%) of the clinicians surveyed reported an understanding of correct recommendations. The second cycle found that only 14 (35%) did not receive driving advice in their discharge letter, and 17 (43%) were given correct advice. Of the contactable patients, 13 (76%) reported being provided a leaflet with the outlined trust driving advice. Moreover, 14 (82%) patients received verbal advice from a healthcare professional. Finally, 27 (90%) of the surveyed clinicians reported understanding the correct advice.

Conclusions

Good targeted education of the residents and consultant clinicians regarding correct driving advice following inguinal hernia repair, as well as visual memory aids in surgical wards, increases the distribution of such advice upon discharge, improving postoperative condition, patient safety, and overall road safety as a result.

## Introduction

Approximately 78,733 surgical inguinal hernia repairs are performed in England each year ​[1]. There are safety and socioeconomic implications to incorrect driving advice following surgery. In the United Kingdom, travelling by car is the most common mode of transport, with 58% of all journeys made by car ​[2].​ Driving is, therefore, an essential part of life for many patients, and abstaining for long periods of time is likely to have work, social, and follow-up implications. Factors that should be considered when advising patients on driving post-inguinal hernia repair include medicolegal literature, research evidence, possible transient femoral nerve palsy, and the effects of opiate medication ​[3].

However, driving too soon following inguinal hernia repair has safety implications. A previous study found reaction time following open and laparoscopic hernia repair to be increased initially, but it returned to the pre-surgery level after one week. In this study, patients who had undergone tension-free endoscopic or open inguinal hernia repair were seated in a car seat before a video display unit to have hand and foot responses to a change in color on a video screen timed on day one, three, and six following surgery. In the endoscopic repair group, the mean hand and foot reaction time was reduced postoperatively on all three days. In the open repair group, mean foot reaction time was slowed on day one (31 ms) and three (32 ms), but was faster than the preoperative period on day six (-7 ms). The increase in the open group on days one and three represented an increase of 9.4% and 9.7%, respectively ​[4]. Therefore, driving within this time frame would increase the risk of road traffic collisions and resulting trauma. This study discussed that abstaining from driving for one week following inguinal hernia repair would be appropriate.

It has been previously noted that advice given following groin hernia repair is variable between surgeons and institutions ​[5].​ A questionnaire published in 2000 found that of 126 surgeons surveyed, advice ranged from driving the same day (three surgeons surveyed) to waiting six to eight weeks (nine surgeons), though the most common response found from this questionnaire was advising patients to wait two weeks following surgery to drive (37 surgeons). In total, 85 of the surgeons surveyed relied on common sense and traditional practice as the source for their advice, with only 16 using published data as an evidence base. Ismail et al. discussed the need for evidence-based national guidelines to be produced and the need for written and verbal advice to be given and documented ​[1].​

The Royal College of Surgeons of England (RCS) has now provided advice to patients following inguinal hernia repair: “Normally, you should refrain from driving for at least one week after a hernia repair operation. You should be free from the distracting effect of pain or the sedative or other effects of any pain relief medication you are taking. You also need to be free of any physical restrictions due to your operation, be comfortable in the driving position and be able to safely control your car, including freely performing an emergency stop” [6,7]. The European Hernia Society guidelines on the treatment of inguinal hernia in adult patients provide no official guidance on driving post-inguinal hernia repair, but note the disparity between surgery and references a study demonstrating that, after seven days, a normal emergency stop response time was achieved in 82% of those who underwent laparoscopic repair and 64% of those who underwent Lichtenstein repair [8]. No specific driving advice is offered by the HerniaSurge group’s International guidelines for groin hernia management [9].

This quality improvement project (QIP) aimed to ensure that University Hospitals of Leicester (UHL) surgeons are able to provide consistent, evidence-based advice on driving following open/laparoscopic elective inguinal hernia repair according to the Trust and RCS advice.

## Materials and methods

Study design and setting

This single-center, retrospective, closed-loop clinical audit was conducted within the Department of General Surgery at the Leicester Royal Infirmary, UHL.

Study period

The initial audit cycle covered cases performed between 09/06/2024 and 11/09/2024, followed by a re-audit conducted from September 2024 to November 2024.

Inclusion and exclusion criteria

All adult male and female patients undergoing elective inguinal hernia repair during the study periods were included. Both unilateral and bilateral procedures were eligible, as were open and laparoscopic approaches, as well as both reducible and irreducible hernias. Patients undergoing emergency inguinal hernia repair were excluded.

First audit cycle

A total of 42 patients, all of whom underwent elective inguinal hernia repair between 09/06/2024 and 11/09/2024, were identified from the UHL trust database of surgical operating lists in the Department of General Surgery. Data were collected manually by members of the surgical team through a review of discharge letters on Nervecentre to determine whether driving advice had been documented, and whether this advice was accurate, defined as recommending one week of driving abstinence and confirmation that the patient could safely perform an emergency stop.

In addition, a telephone survey was conducted to assess whether patients had received driving advice verbally or via written information (leaflets). The patients were asked the following specific questions during this survey: “Did you receive any driving advice during your discharge from the hospital?” “If yes, what advice were you given during that time?” “Did you receive any verbal advice?” “Did you receive any leaflet/written advice?” and “Were you involved in any RTC after your operation?”

A separate survey of 51 clinicians was conducted using Google Forms (Google Inc., USA) to evaluate awareness and consistency of driving advice provided postoperatively. The following questions were asked: “Do you understand the current trust recommendations regarding driving advice during discharge of the patients who had elective inguinal hernia repair?” “Did you give any advice on driving after the procedure?” “Did you give any verbal advice?” “Did you give patients any written advice?” and “Did you provide patients with any leaflets?”

Re-audit cycle

The re-audit included 40 patients undergoing elective inguinal hernia repair between September and November 2024. A total of 30 clinicians were surveyed using the same Google Forms questionnaire. A further telephone survey was conducted, and 17 patients were contactable and consented to discuss any written or verbal postoperative driving advice they had received, based on the same questions asked during the first cycle.

Data presentation

**Figure 1:**
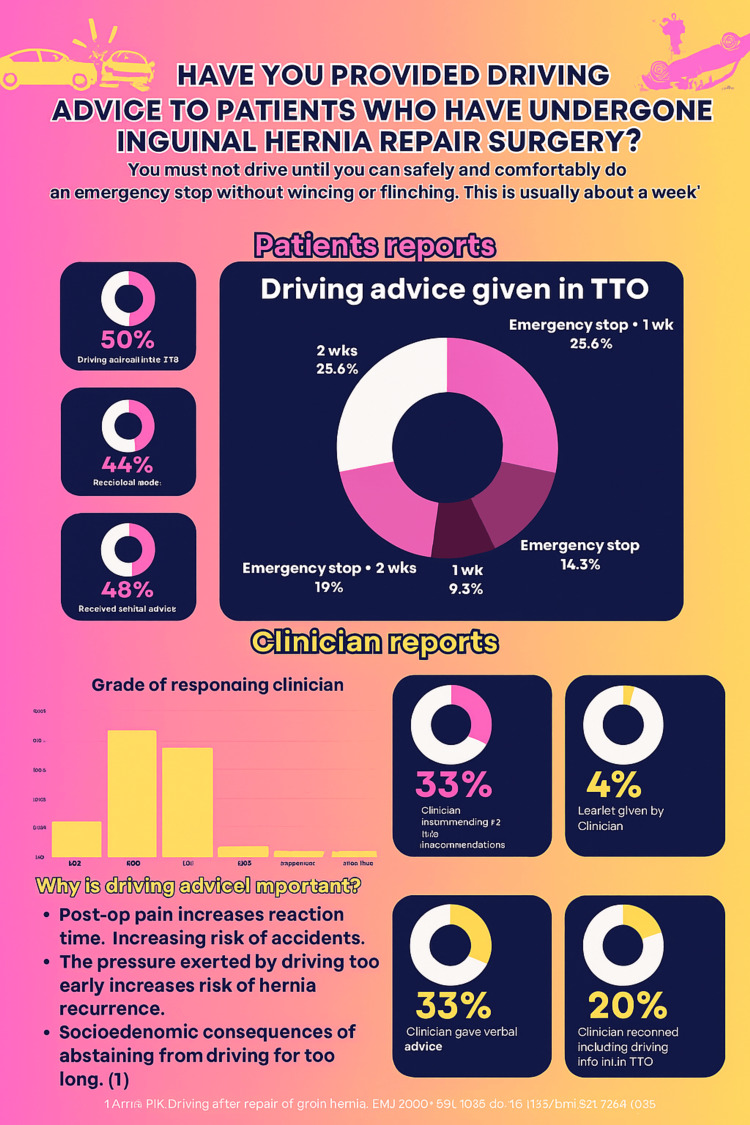
Poster display as a memory aid to encourage clinicians to include driving advice in the TTOs. Image credit: Ms. Anika Tahsin (author).

**Figure 2:**
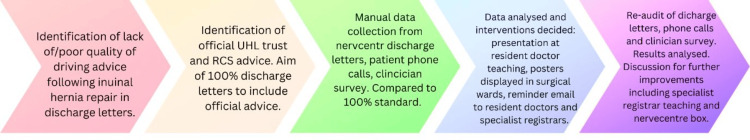
Schematic flowchart of data collection. Image credit: Ms. Anika Tahsin (author).

Findings from both audit cycles were summarized and presented in poster format (Figure 1). The data collection process is illustrated in a flowchart (Figure 2).

## Results

**Figure 3:**
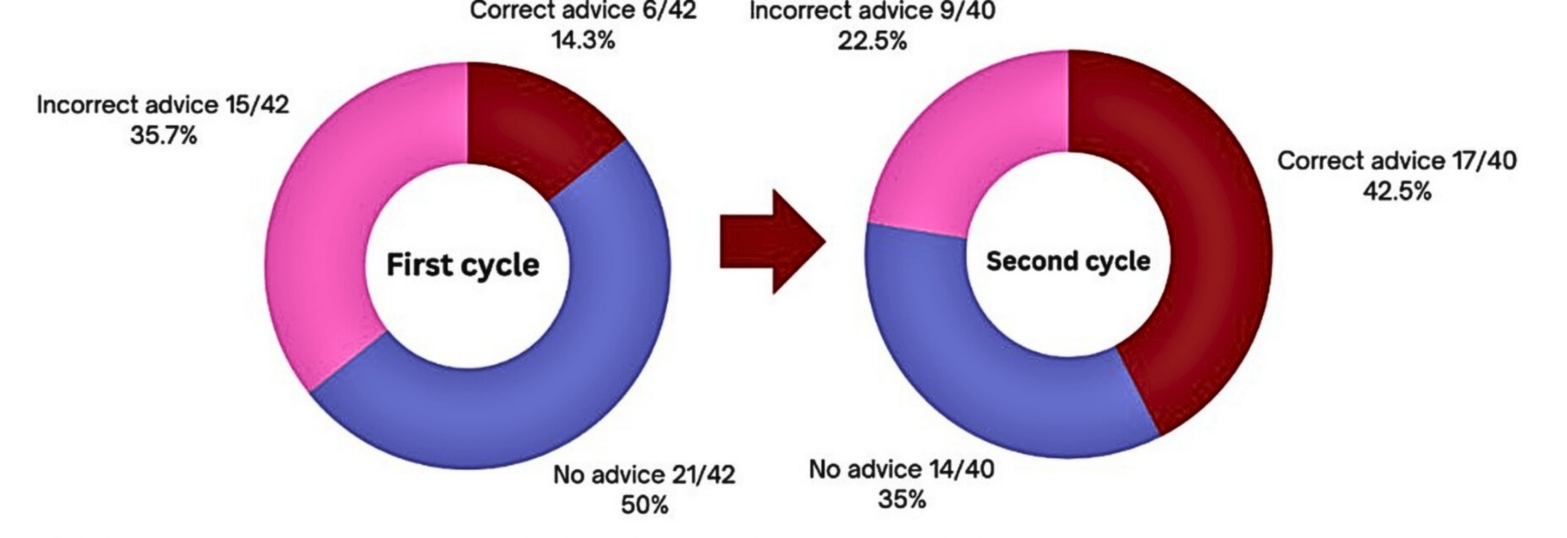
Patients undergoing inguinal hernia repair surgery receiving driving advice as per the University Hospitals of Leicester and Royal College of Surgeons of England guidelines in hospital discharge letters.

The first cycle consisted of 42 patients post-inguinal hernia surgery, among whom 37 (88%) were male and five (12%) were female. The median age of patients was 64 years (ranging from 22 to 90). On review of 42 patient TTOs, 21 (50%) did not receive any driving advice, six (14.3%) received correct advice, and 15 (35.7%) received incorrect advice (Figure 3).

Following intervention, a second cycle of 40 patients post-inguinal hernia surgery was conducted, among whom 38 (95%) were male and two (5%) were female. The patients had a median age of 67.5 years (ranging from 18 to 84), slightly higher than the previous cycle. Of the 40 patients identified, 14 (35%) did not receive driving advice in their discharge letter; however, this was an improvement from the first cycle. Overall, 17 (42.5%) were given correct advice, and nine (22.5%) patients received incorrect driving advice (Figure 3), both of which were an improvement.

**Figure 4:**
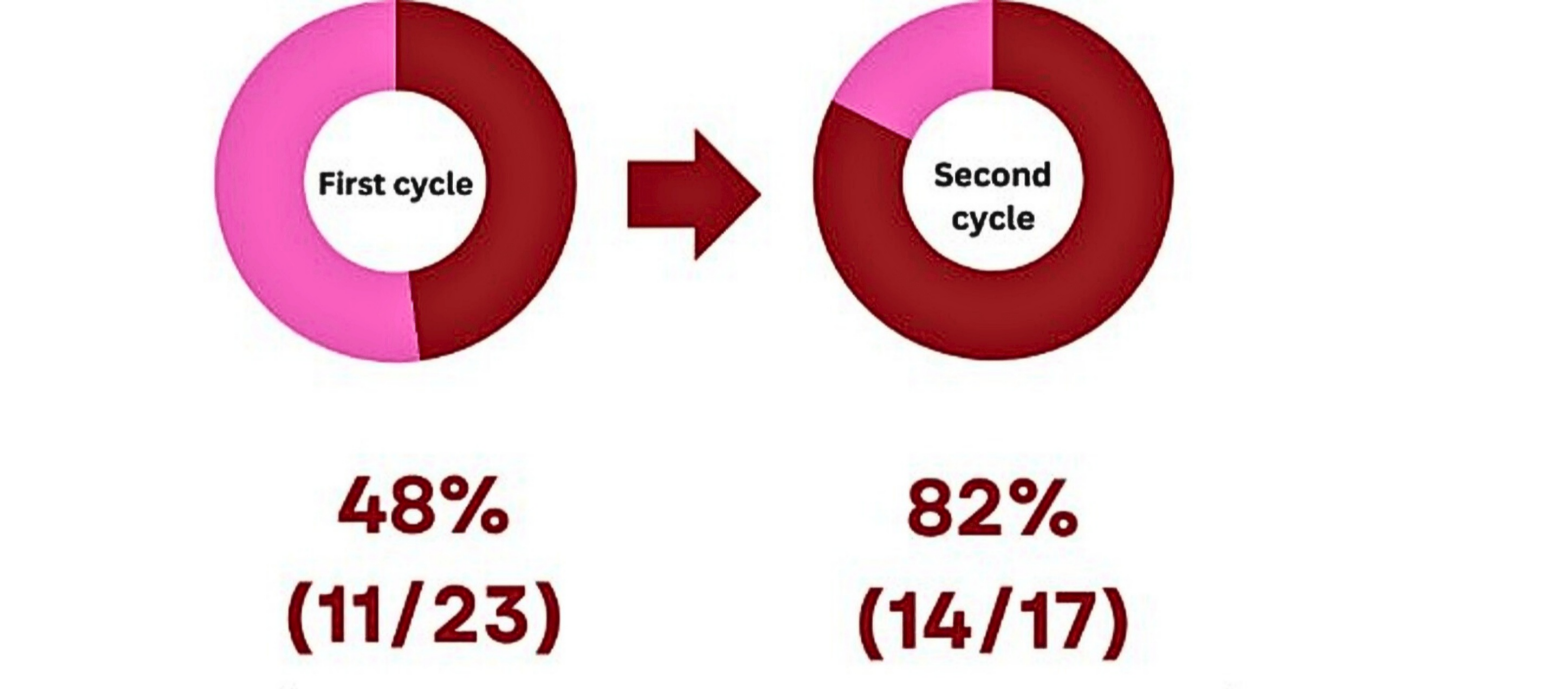
Patients who were traceable using contact numbers confirmed receiving verbal advice regarding safe driving from operating surgeons. Red: Patients receiving verbal advice. Pink: Patients not receiving verbal advice.

During the first cycle, where 23 patients were contactable via telephone, 10 (43%) of these 23 patients received written advice in the form of a leaflet. Overall, 11 (48%) patients received verbal advice from a healthcare professional (Figure 4). Moreover, none (0%) of the 42 patients were involved in any road traffic collisions following their operation, up until the point of contact.

During the second cycle telephone survey of 17 patients, 13 (76%) reported being provided a leaflet with the outlined trust driving advice. Overall, 14 (82%) patients received verbal advice from a healthcare professional (Figure 4). Moreover, none (0%) were involved in any road traffic collisions up until the point of contact.

**Figure 5:**
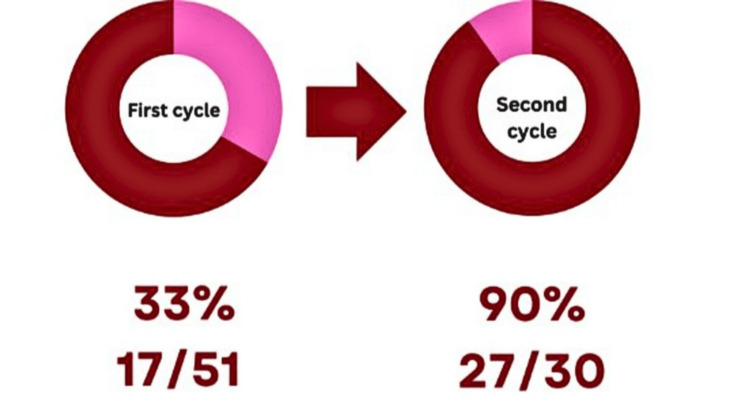
Clinicians (FY 1-2, CT 1-2, ST 1–8) in general surgery team reporting understanding the recommendations. Red: Percentage of clinicians who understood. Pink: Percentage of clinicians who did not understand.

In total, 51 clinicians responded to the Google Forms during the first cycle, of whom 17 (33%) declared they understood the current trust recommendations. A total of 30 clinicians completed the form in the second, with 27 (90%) reporting a good understanding of the current trust guidance (Figure 5).

**Figure 6:**
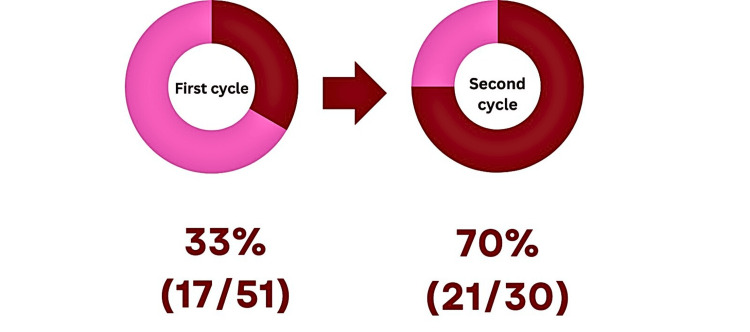
Clinicians reporting giving verbal driving advice. Red: Clinicians who gave verbal advice. Pink: Clinicians who did not give verbal advice.

Among the 51 clinicians who completed the questionnaire, 17 (33%) admitted that they gave verbal advice to patients following hernia repair in the first cycle, whereas in the second cycle, it improved dramatically; 21 (70%) out of 30 clinicians were found to give verbal advice (Figure 6).

**Figure 7:**
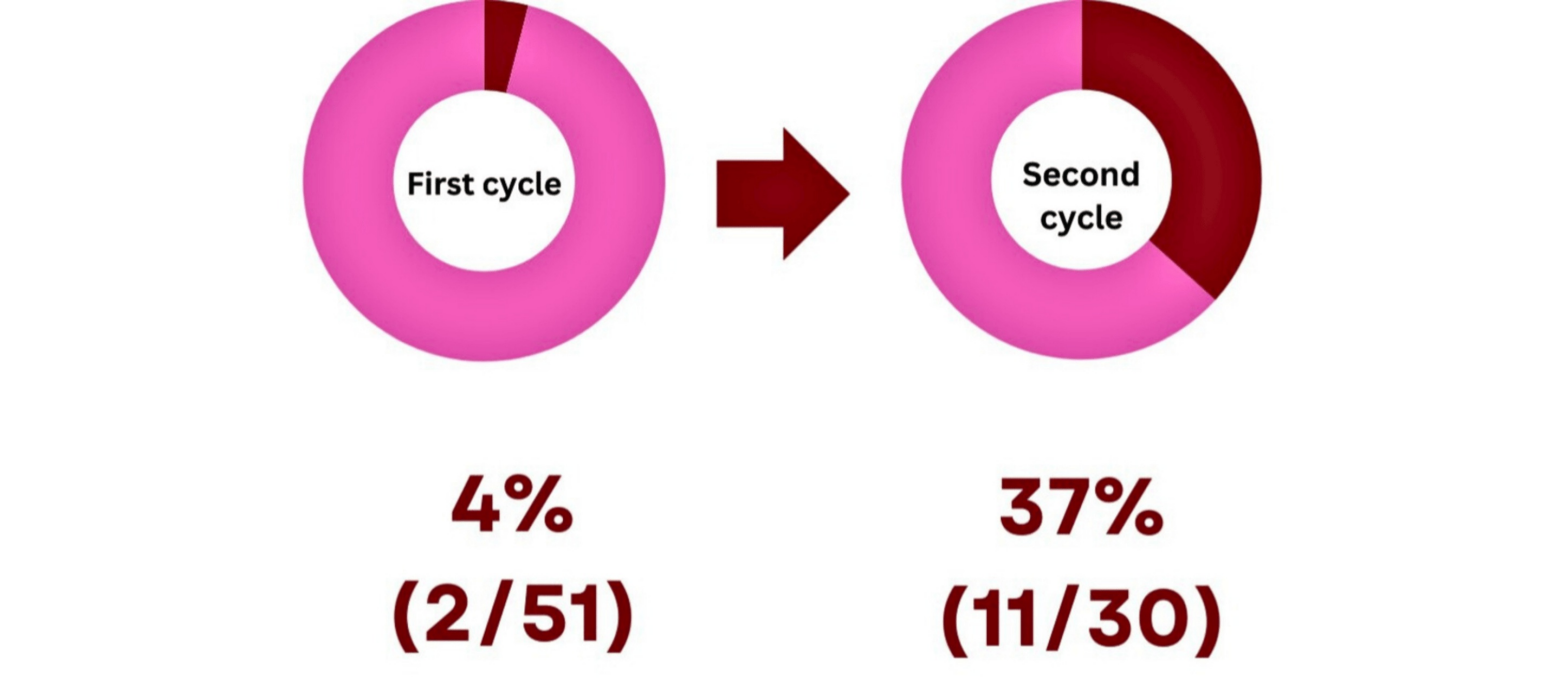
Clinicians reporting distributing leaflets. Red: Clinicians who distributed leaflets. Pink: Clinicians who did not distribute leaflets.

Only two (4%) clinicians gave leaflets to patients during the first cycle, which improved significantly in the second cycle; 11 (37%) clinicians reported distributing leaflets to patients (Figure 7).

## Discussion

Following the intervention, the recipients of correct driving advice in the discharge summary post-elective inguinal repair improved from six (14%) to 17 (43%). Educating both the foundation doctors and specialist trainee doctors who most commonly write patient TTOs has been shown to be of benefit and ultimately improves patient safety.

Not only did the frequency of driving advice in the TTO improve by 15% but the accuracy also improved by an additional 29% with far fewer patients receiving incorrect driving advice. Improving clinicians’ knowledge of the correct driving advice will allow patients to be better informed and remain safer on the roads. This was highlighted when contacting patients, several of whom had waited significantly longer than necessary due to incorrect verbal advice provided. This included, on several occasions, a delay of six or more weeks. The number of patients who received verbal and leaflet advice improved post-intervention, with a 34% and 33% improvement, respectively. Reassuringly, both cycles reported no road traffic collisions.

In parallel with the discharge letter and patient data, clinician reports also improved significantly. A 57% increase in clinician understanding of the trust recommendations was encouraging, likely due to face-to-face and online teaching sessions outlining the driving advice in the form of lectures performed by the author, and reminder emails to them from the author, saying “Please do not forget to mention driving advice during discharge of the patients who underwent elective inguinal hernia repairs. Please include both verbal and written forms of advice.” Repeating this teaching for other rotating foundation doctors would be a vital continuation. Teaching sessions focusing on other surgeries that may have differing or additional driving advice would be helpful. Further actions, such as repeated teaching sessions, may be required to ensure that 100% of discharge letters have driving advice included, negating the risk of any potential safety issues upon discharge. Not only will the driving advice reduce rates of road traffic collisions, but it will also reduce postoperative complications such as infection rate, postoperative pain, hematoma, recurrence of hernia, and wound dehiscence.

Amid’s 2000 editorial on driving advice following groin hernia repair ​outlined the risks of driving soon after hernia repair [10]. Given that the edges of a surgical defect take between six to eight weeks to repair, a sudden increase in intra-abdominal pressure may disrupt these suture lines, leading to wound dehiscence and prolonged complex recovery [11]. Avoiding high-speed collisions is vital. Encouraging patients to practice emergency stops in a stationary car to assess the pain threshold may be an appropriate level of future guidance. This would be similar to the RCS guidance on driving advice following laparoscopic cholecystectomy and nephrectomy​ [12,13].

Postoperative pain remains the key factor in returning to driving, with prolonged pain resulting in a declining psychological state. This results in its own problems, including limited daily functioning and a declining quality of life, while paradoxically amplifying pain over time. It is essential to manage postoperative pain, allowing patients to maintain their independence and continue a positive recovery [14].

Improving clinician knowledge of driving advice is vital not just to inguinal hernia surgery but to all surgeries. Introducing some changes in the IT system, such as a spreadsheet or infographic outlining the differing driving advice following all major surgeries, would be a useful tool for surgical clinicians. This would allow for safe and appropriate advice given both in the TTOs and verbally to patients. Interestingly, the length and complexity of surgery have been shown to impact the frequency of road traffic accidents following surgery [8,15]. Being able to provide specific advice to patients who underwent more challenging procedures may allow for more individual recovery regimes.

Further improvements to this study may include offering set leaflets to patients attending inguinal hernia repair clinics rather than the advice buried within the information pack. Patients reported the information pack being too detailed and therefore chose not to read all the advice. Offering these preoperatively adds another step in the process of patient education. Educating both resident doctors and registrars is vitally important, as it is often the registrars who write the discharge letters in day case surgeries. Maintaining regular teaching/e-learning sessions, especially for newly rotating resident doctors, is imperative. A change in the Nervecentre discharge summary section seems an appropriate advancement, allowing clinicians a prompt to include driving advice and other potential postoperative surgical advice, such as “Lifting heavy objects and sports advice.”

Limitations

The primary limitation of this audit remains the small sample size. Unfortunately, only doctors were surveyed during clinician reports, which excludes potential other staff, most notably physician assistants, who often complete the discharge summaries. We also had to assess their understanding of trust recommendations on driving advice and verbal advice giving by a simple questionnaire, which generated a risk of a potential self-report bias. A future survey, after including the other team members and creating a more detailed questionnaire, may provide a more thorough insight into clinician education. Moreover, due to the retrospective approach, we were unable to reach all patients when contacting them via phone. This reduced the sample size even further when gathering leaflet and verbal advice data and made it prone to selection bias. In the future, we hope this audit may be utilized on a larger scale, as it only gives a focused regional picture. Repeating at a national scale would give a more precise idea of the changes required.

## Conclusions

Good education of clinicians has been shown to significantly improve the driving advice given to patients following inguinal hernia repair. Both verbal and leaflet advice, and, most importantly, clinician understanding improved following the intervention. We would seek to continue improvement of driving advice given, and further re-auditing, regular teaching sessions, and prompts in the IT system would be recommended to sustain these improvements. This is vital for future patient safety and satisfaction, as well as overall road safety.
